# A nitrogen source-dependent inducible and repressible gene expression system in the red alga *Cyanidioschyzon merolae*

**DOI:** 10.3389/fpls.2015.00657

**Published:** 2015-08-26

**Authors:** Takayuki Fujiwara, Yu Kanesaki, Shunsuke Hirooka, Atsuko Era, Nobuko Sumiya, Hirofumi Yoshikawa, Kan Tanaka, Shin-Ya Miyagishima

**Affiliations:** ^1^Department of Cell Genetics, National Institute of GeneticsMishima, Japan; ^2^NODAI Genome Research Center, Tokyo University of AgricultureTokyo, Japan; ^3^Japan Science and Technology Agency, Core Research for Evolutional Science and TechnologyKawaguchi, Japan; ^4^Department of Bioscience, Tokyo University of AgricultureTokyo, Japan; ^5^Chemical Resources Laboratory, Tokyo Institute of TechnologyYokohama, Japan; ^6^Department of Genetics, Graduate University for Advanced StudiesMishima, Japan

**Keywords:** algae, plants, homologous recombination, conditional knockdown, gene inducible system

## Abstract

The unicellular red alga *Cyanidioschyzon merolae* is a model organism for studying the basic biology of photosynthetic organisms. The *C. merolae* cell is composed of an extremely simple set of organelles. The genome is completely sequenced. Gene targeting and a heat-shock inducible gene expression system has been recently established. However, a conditional gene knockdown system has not been established, which is required for the examination of function of genes that are essential to cell viability and primary mutant defects. In the current study, we first evaluated the expression of a transgene from two chromosomal neutral loci located in the intergenic region between CMD184C and CMD185C, and a region upstream of the *URA5.3* gene. There was no significant difference in expression between them and this result suggests that both may be used as neutral loci. We then designed an inducible and repressible gene expression by using promoters of nitrate-assimilation genes. The expression of nitrate-assimilation genes such as *NR* (nitrate reductase), *NIR* (nitrite reductase), and *NRT* (the nitrate/nitrite transporter) are reversibly regulated by their dependence on nitrogen sources. We constructed stable strains in which a cassette containing the *NR, NIR*, or *NRT* promoter and *sfGFP* gene was inserted in a region upstream of *URA5.3* and examined the efficacy of the promoters. The *NR, NIR*, and *NRT* promoters were constitutively activated in the nitrate medium, whereas their activities were extremely low in presence of ammonium. The activation of each promoter was immediately inhibited within a period of 1 h by the addition of ammonium. Thus, a conditional knockdown system in *C. merolae* was successfully established. The activity varies among the promoters, and each is selectable according to the expression level of a target gene estimated by RNA-sequencing. This method is applicable to defects in genes of interest in photosynthetic organism.

## Introduction

In studies on photosynthetic eukaryotes, molecular genetic techniques have been developed and used extensively for certain land plants and the unicellular green alga *Chlamydomonas reinhardtii* widely used as model systems. For the investigation of phenomena that are generally shared by algae and land plants, unicellular algae offer several experimental advantages. For example, a relatively homogeneous population is available in unicellular algae, in contrast to land plants, in which cells differentiate into heterogeneous populations. The generation time of unicellular algae is much shorter than that required for multicellular land plants. Thus far, *C. reinhardtii* has been the most extensively studied green alga, because it is genetically tractable. However, overexpression of a given gene of interest and expressing the genes of other organisms are very difficult because of the silencing of transgenes (Rosales-Mendoza et al., 2012; Liu and Benning, 2013). In certain other microalgae, methods for transformation have been reported, but as yet are still far from having come into practical use (Gong et al., 2011).

The unicellular red alga *Cyanidioschyzon merolae* inhabits sulfate-rich hot springs. The cell organization is very simple and the cell contains a single nucleus, mitochondrion and chloroplast along with a minimum set of membranous structures. The cell also has simple architectures of the endoplasmic reticulum and Golgi body, a single peroxisome and a small number of lysosomes (vacuoles; Kuroiwa et al., 1998; Misumi et al., 2005). The cell division and organelle division are tightly synchronized by the light/dark cycle (Suzuki et al., 1994). The nuclear, chloroplast, and mitochondrial genomes have been completely determined (i.e., without any gaps; Ohta et al., 1998, 2003; Matsuzaki et al., 2004; Nozaki et al., 2007). It possesses an extremely simple nuclear genome (16.5 Mbp; 4,775 protein-coding genes) among the photosynthetic eukaryotes (c.f. the green alga *C. reinhardtii*, 120 Mb; the land plant *Arabidopsis thaliana*, 157 Mb) with low genetic redundancy (Matsuzaki et al., 2004; Misumi et al., 2005; Nozaki et al., 2007). Therefore, this alga is suitable for various “omics” analyses. Various methods for genetic manipulation have been developed, such as the transient expression of proteins from plasmids (Ohnuma et al., 2008, 2009, 2014; Watanabe et al., 2011) and gene targeting by homologous recombination (Minoda et al., 2004; Imamura et al., 2010).

By virtue of a combination of these salient features and the experimental techniques of molecular genetics, *C. merolae* has become a promising model organism for the study of cell biology and metabolism in photosynthetic eukaryotes, such as chloroplast and mitochondrial division (Miyagishima et al., 2001, 2003; Nishida et al., 2003, 2007; Yoshida et al., 2009, 2010), vacuolar inheritance (Yagisawa et al., 2007, 2009; Fujiwara et al., 2010), the dynamics of other organelles (Miyagishima et al., 1998; Yagisawa et al., 2012, 2013; Fujiwara et al., 2013a; Imoto et al., 2013), nitrogen assimilation (Imamura et al., 2008, 2010) and circadian rhythms (Miyagishima et al., 2014). However, to study the mechanisms underlying the activities that are essential to cellular growth and survival, inactivation or modification of the mechanisms by gene manipulation would likely be lethal. Even when a certain gene manipulation does not lead to cell death, it is likely that prolonged cultivation of such genetically modified cells would lead to secondary indirect defects in the cells, which makes it extremely difficult to evaluate any primary defects that are directly caused by the gene manipulation.

To overcome this critical problem, analyses of primary defects, using conditional knockdown, an approach which is not effective under permissive environmental conditions but can be effective under certain tightly restricted conditions, are greatly desired. In contrast to *C. reinhardtii*, in which the expression of transgenes is often silenced, transgenes (either endogenous or exogenous genes) are stably expressed in *C. merolae* (Fujiwara et al., 2013b; Sumiya et al., 2014; Watanabe et al., 2014), which is an important advantage for constructing a gene-regulation system. To this end, an inducible gene expression system using the heat-shock promoter has recently been developed in *C. merolae* (Sumiya et al., 2014). This heat-shock system enables an examination of the effect of the expression of modified genes (e.g., dominant negative forms of the protein) on the cells, but it is difficult to evaluate the effect of repression of gene expression in this system. This is because culturing at such a high temperature (50°C) for a long period, which is required to maintain the expression of a target gene, damages cells and ultimately leads to cell lethality before the repression of the gene expression. Thus, an inducible/repressible gene expression system based on a less severe environmental condition is desired, as in the case in budding yeast, in which the regulatory systems for gene expression have been developed by using either carbon-source dependent promoters or a tetracycline-inducible/repressible system (Kaufmann and Knop, 2011).

To develop an inducible/repressible gene expression system, we focused on the promoters of the nitrate assimilation genes in *C. merolae* based on the following characteristics. A large number of eukaryotes and prokaryotes assimilate nitrogen from ammonium and nitrate (Ohmori et al., 1977; ter Schure et al., 2000). In many cases, ammonium is preferred over nitrate as an inorganic nitrogen source, because nitrate must be reduced to ammonium by expending energy for the purpose of its assimilation (Warner and Kleinhofs, 1992). When the balance between ammonium and nitrate changes in the environment, cell metabolism changes in order to adapt (Engelsberger and Schulze, 2012). Previous transcriptome analyses in *C. merolae* showed that transcription of certain key nitrogen assimilation genes is induced when the cells are transferred from the nitrogen (ammonium)-replete medium to the nitrogen-depleted medium (Imamura et al., 2008). The induced genes include *NRT* (the nitrate/nitrite transporter, CMG018C, the gene number is taken from http://merolae.biol.s.u-tokyo.ac.jp/), *NR* (nitrate reductase, CMG019C), *NIR* (nitrite reductase, CMG021C), *AMT* (the high affinity ammonium transporter, CMT526C), and *GS* (glutamine synthetase, CMI233C; Imamura et al., 2008).

In this study, we first confirmed that at least two chromosomal loci are sufficient to express a transgene as neutral loci and then developed a nitrogen-source-dependent inducible/repressible gene expression system by using the promoters of nitrate-assimilation genes.

## Results

### Estimation of an Ability to Express Transgene of Two Different Neutral Loci by Comparing sfGFP Expression

In order to evaluate the activities of promoters of nitrate-assimilation genes, we planned to express reporter protein [green fluorescent protein (GFP)] under the control of respective promoters from a *C. merolae* neutral chromosomal locus.

We have used the convergent intergenic region of CMD184C and CMD185C as a neutral locus because it is very short and therefore not likely to contain promoter activities that would potentially affect gene expression in its vicinity (Fujiwara et al., 2013b). However, it has not been determined whether the intergenic region is effective compared to other putative neutral loci. Recently, Watanabe et al. integrated a *GFP* gene in the vicinity of the chromosomal *URA5.3* locus (CMK046C) and succeeded in expressing the GFP protein (Watanabe et al., 2014). Based on that report, we first compared the two chromosomal loci, namely the intergenic region between CMD184C and CMD185C and the upstream of *URA5.3* locus, at the level of *GFP* transgene expression.

**FIGURE 1:**
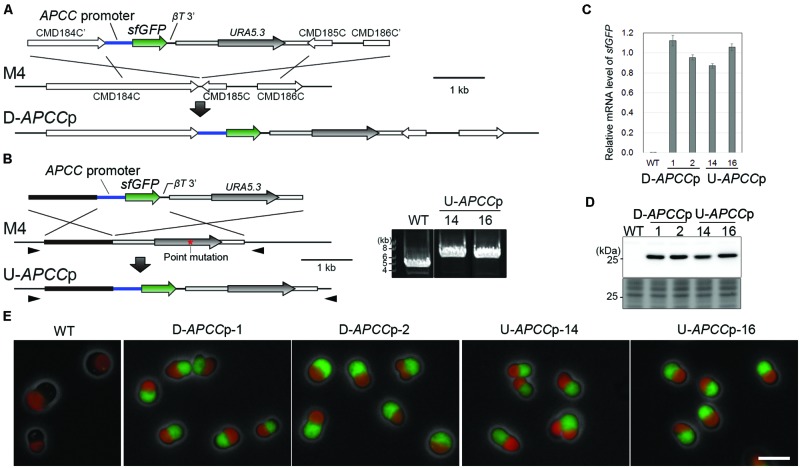
**Construction and characterization of stable strains expressing sfGFP from two distinct chromosomal neutral loci. (A)** Schematic diagram of the *sfGFP* gene insertion into the intergenic region between CMD184C and CMD185C by homologous recombination. The first line indicates the introduced DNA fragment and the second line indicates the genomic structure of the parental strain M4. For the efficient expression of *sfGFP*, the 600-bp upstream flanking sequence of *APCC orf* (*APCC* promoter) and the 200-bp downstream flanking sequence of the β-tubulin *orf* (βT 3′) were utilized as a promoter and a putative polyadenylation signal sequence, respectively. The third line indicates the expected genomic structure of the stable transformant. **(B)** Schematic diagram of the *sfGFP* gene insertion into the upstream region of the *URA5.3* gene by homologous recombination. The same set of the promoter and the putative polyadenylation signal sequence as in **(A)** was flanked with the *sfGFP orf*. The red asterisk indicates the position of a point mutation in *URA5.3* gene in the strain M4. The arrowheads indicate the positions of the PCR primers No. 14 and No. 15 (the exact positions and sequences are indicated in Supplementary Table S1). The PCR analysis of the independent U-*APCC*p strains confirmed the homologous recombination events (right panel). The WT strain was used as a negative control. The predicted size of the PCR product is 5.9 kb for U-*APCCp* and 4.2 kb for the WT, respectively. **(C)** Quantitative RT-PCR analysis of the D-*APCC*p and U-*APCC*p strains to estimate the *sfGFP* mRNA levels. The WT strain was used as a negative control. The *sfGFP* mRNA level in each strain was normalized with the data of *DRP3*/*CmDnm1*, and the average of the values in the D-*APCC*p and U-*APCC*p strains was defined as 1.0. The bar indicates the standard deviation (*n* = 3). **(D)** Immunoblot analysis of the total cell lysates with the anti-GFP antibody. An image of a gel stained with Coomassie Brilliant Blue (CBB) is shown as a loading control. **(E)** Fluorescent micrographs showing sfGFP fluorescent signals detected in the D-*APCC*p and U-*APCC*p strains. Green, GFP; red, autofluorescence of chlorophyll; gray, phase-contrast. Scale bar = 5 μm.

We previously prepared a strain in which the *APCC* promoter*-superfolder GFP* (*sfGFP*) and *URA5.3* genes are integrated into the intergenic region between CMD184C and CMD185C of the *C. merolae* uracil-auxotrophic strain M4, which possesses a point mutation in the chromosomal *URA5.3* locus (Sumiya et al., 2014; termed D-*APCC*p in this study; **Figure 1A**). For comparison, we prepared a strain in which the *APCC* promoter*-sfGFP* and *URA5.3* selection genes are integrated into the chromosomal *URA5.3* locus of M4. In this strain, termed U-*APCC*p, the *APCC* promoter*-sfGFP* is integrated into the region upstream of the *URA5.3* locus (**Figure 1B**, the left panel). The occurrence of the recombination events in an upstream region of the *URA5.3* locus in the U-*APCC*p strains were confirmed by PCR (**Figure 1B**, right panel).

In order to estimate the level of transgene expression at both neutral loci, we compared the mRNA and protein levels along with the fluorescent intensity of sfGFP between the D-*APCC*p and U-*APCC*p strains. Quantitative reverse transcription polymerase chain reaction (qRT-PCR) assay and immunoblotting showed that almost the same levels of the *sfGFP* mRNA and sfGFP protein were expressed in the two strains (**Figures 1C,D**). By fluorescence microscopy, a similar level of GFP fluorescence was detected in D-*APCC*p and U-*APCC*p cells (**Figure 1E**). These results indicate that there is no significant difference in the ability to express a transgene between the two chromosomal loci and suggest that both may be used as neutral loci for the expression of transgenes.

### The Activation and Strength of Activity of the *NR, NIR*, and *NRT* Promoters for the Expression of the *sfGFP* Transgene in the Nitrate Medium

**FIGURE 2:**
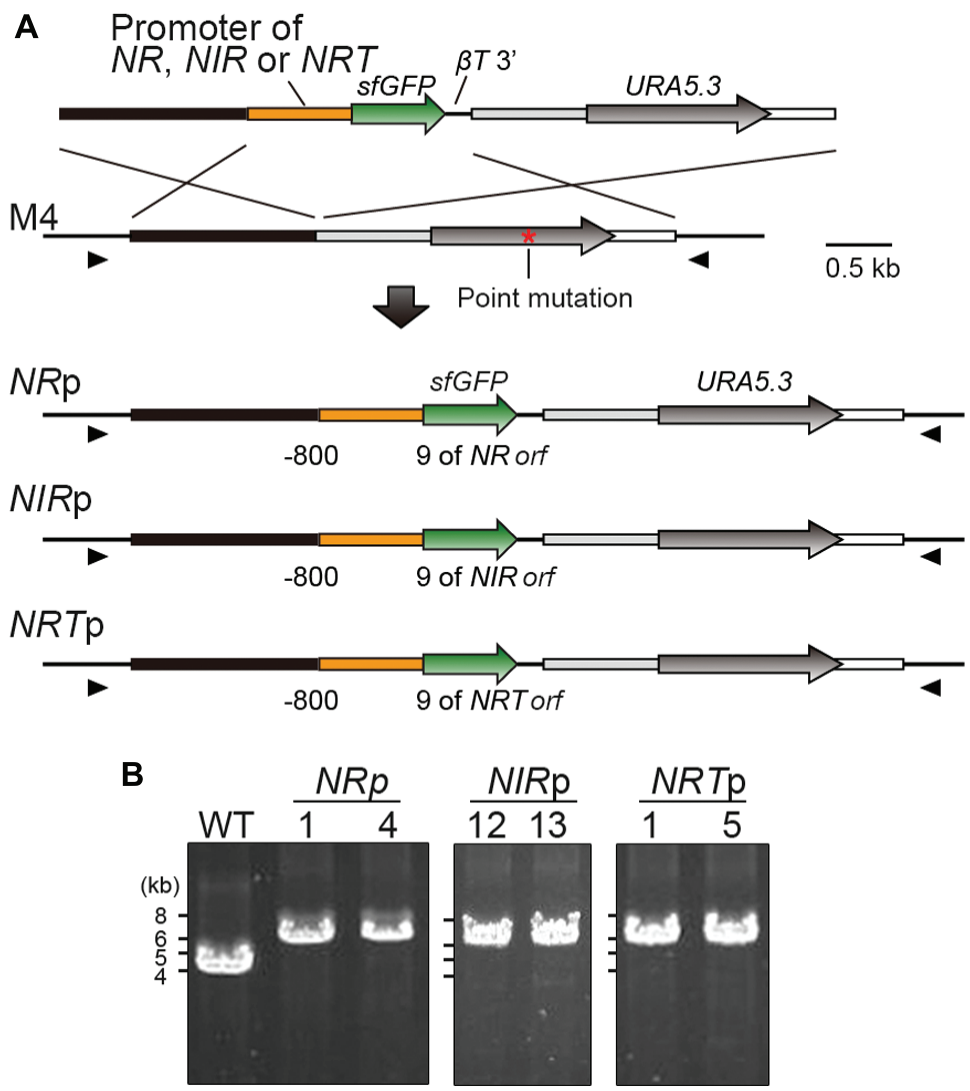
**Construction of transformants expressing sfGFP by using the promoters of the nitrate assimilation genes. (A)** Schematic diagram showing the targeted insertion of *sfGFP* flanked with the *NR, NIR*, or *NRT* promoter into the chromosomal region upstream of the *URA5.3* gene. The third to fifth lines indicate the expected genomic structures in which a single copy of the transformed sequence is inserted by double-crossover homologous recombination in the respective cases. The red asterisk shows the position of the point mutation in the *URA5.3* gene in strain M4. The arrowheads indicate the positions of the PCR primers used in **(B)**. **(B)** PCR analysis of independent transformants confirmed the homologous recombination events. The WT strain was used as a negative control. The genomic sequence of the WT strain is identical to that of M4 except for the point mutation in the *URA5.3* gene in M4. The predicted size of the PCR product is 6.1-kb for *NR*p, *NIR*p, and *NRT*p or 4.2 kb for the WT, respectively. The positions and sequences of the primers No. 14 and No. 15 are shown in Supplementary Table S1.

Among *NR, NIR, NRT, AMT* and *GS*, the expression of which were induced by ammonium-depletion in a previous study (Imamura et al., 2008), we focused on the promoters of *NR, NIR* and *NRT*, because *AMT* and *GS* exhibited leaky transcription in ammonium-replete medium (Imamura et al., 2008). To test the effectiveness of the promoters in turning the expression of transgenes on or off, we integrated the *NR, NIR*, or *NRT* promoter (the 800-bp upstream flanking sequence of the respective genes) -*sfGFP orf* into the upstream region of the chromosomal *URA5.3* locus in the same manner as in the U-*APCC*p strain (**Figure 2A**). The recombination events in the upstream region of *URA5.3* locus was confirmed by PCR with primers No. 14 and No. 15 (**Figure 2B**). The resultant transformants were termed *NR*p, *NIR*p, and *NRT*p. The *NR*p-1, *NIR*p-12, and *NRT*p-1 strains were used for further investigation.

In order to activate the nitrate-assimilation gene promoters, *NR*p, *NIR*p, and *NRT*p cells in logarithmic culture were transferred from the ammonium medium to the nitrate medium.

**FIGURE 3:**
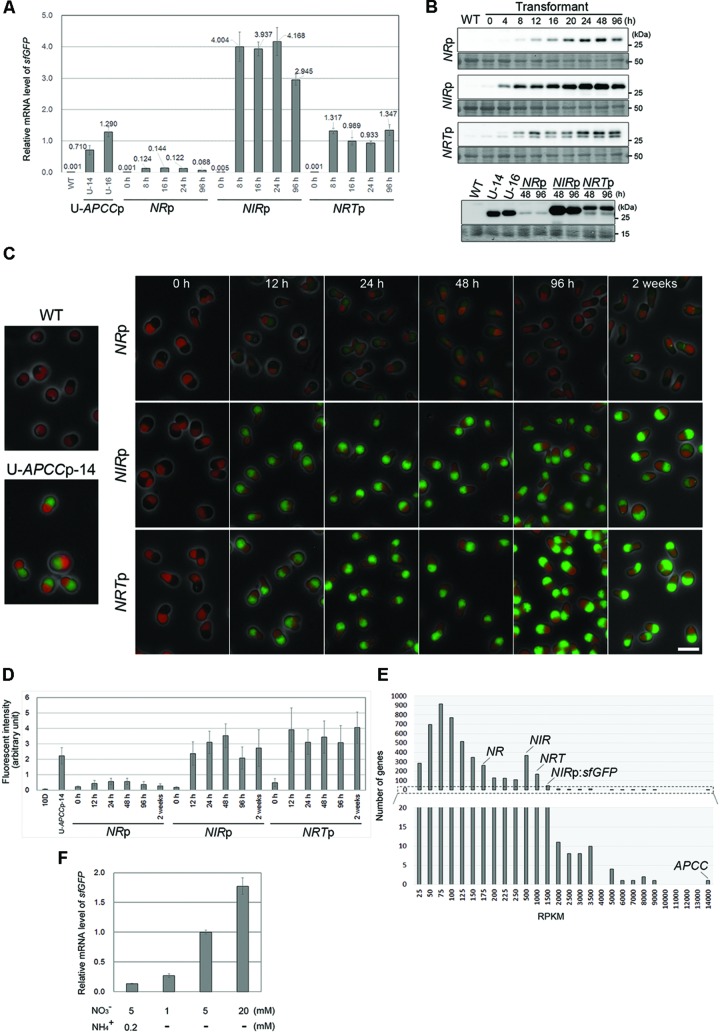
**The induction of sfGFP expression by medium exchange in the *NR*p, *NIR*p, and *NRT*p strains. (A)** Quantitative RT-PCR analyses showing the change in the *sfGFP* mRNA level in the *NR*p, *NIR*p, and *NRT*p strains before and after the exchange from the ammonium medium to the nitrate medium. The values were standardized by the average of the U-*APCC*p-14 and U-*APCC*p-16 which were cultured in the ammonium medium. The WT strain was used as a negative control. The time elapsed since the medium exchange is indicated below the graph and the time point 0 is just before the medium exchange. The bar indicates the standard deviation (*n* = 3). **(B)** Immunoblot analysis of the total cell lysates of the respective strains with the anti-GFP antibody. The upper panel shows the change in the sfGFP protein level just before (0 h) and after the medium exchange. The WT strain was used as a negative control. The lower panel compares the sfGFP protein level of the respective strains after the medium exchange. The sfGFP level in the U-*APCC*p strains (stably expressing GFP in both the ammonium and nitrate media: the results in the ammonium medium shown) is also shown as an index of the protein level. The WT strain was used as a negative control. An image of the PVDF membrane stained with Ponceau S is shown as a loading control. Two bands were detected in the *NRT*p strain with the anti-GFP antibody, most likely because there are two translational start sites. **(C)** Fluorescent micrographs showing the change in the level of the sfGFP fluorescent signal just before (0 h) and after the medium exchange. The green fluorescence of sfGFP was overlaid with the phase-contrast image and autofluorescence of the chloroplast. The WT and the U-*APCC* strain were used as a positive and a negative control, respectively. The exposure time to capture the sfGFP signal was 1 s for all images. Green, GFP; red, autofluorescence of chlorophyll; gray, phase-contrast. The scale bar = 5 μm. **(D)** The change in the intensity of sfGFP fluorescence in cells in **(C)**. The bar indicates the standard deviation (*n* = 15). **(E)** RNA-seq analysis showing the relative mRNA levels of endogenous *NR, NIR*, and *NRT* in the transcriptome of the *NIR*p strain (logarithmic growth phase) cultured in the nitrate medium. The reads per kilo-base (RPKM) data indicates the relative mRNA abundance. The RPKM of endogenous *NR, NIR, NRT, APCC*, and *sfGFP* transcription of which is driven by the *NIR* promoter, was 158, 377, 787, 13384, and 1337, respectively. The lower histogram shows the magnification of the range from 0 to 20. **(F)** QRT-PCR analyses showing the *sfGFP* mRNA level in the *NRT*p strain 16 h after the medium exchange. The values were standardized by the data from culture in medium containing 5 mM nitrate without ammonium. The concentration of nitrate or ammonium is indicated below the graph. The bar indicates the standard deviation (*n* = 3).

Quantitative reverse transcription polymerase chain reaction revealed that the mRNA level of the *sfGFP* transgene in *NR*p, *NIR*p, and *NRT*p was extremely low before the medium exchange (0 h in **Figure 3A**; Based on the mean *sfGFP* mRNA levels in U-*APCC*p-14 and -16 that were cultivated in the ammonium medium and used as reference values).

After the medium exchange, the *sfGFP* mRNA level increased and reached a maximum in 8 h (**Figure 3A**). The maximum level of s*fGFP* mRNA varied with the promoter used and increased on the order of *NR*p, *NRT*p and *NIR*p, with this last one the highest.

Immunoblot analyses showed that the sfGFP protein was below the detectable level before the medium exchange (0 h). sfGFP accumulated and the level reached a maximum in a period from 24 to 48 h after the medium exchange in each strain (**Figure 3B**, the upper panel). Two bands were detected in the *NRT*p strain with the anti-GFP antibody, likely because there are two translational start sites. When the maximum level of sfGFP was compared, the level was the highest in *NIR*p, followed by *NRT*p, U-*APCC*p and *NR*p (**Figure 3B**, the lower panel), as was also the case for the mRNA level (**Figure 3A**).

Under fluorescence microscopy, sfGFP fluorescence was not detected before the medium exchange (0 h in **Figure 3C**). After the medium exchange, the fluorescent signal became evident and the signal in *NR*p cells was the weakest (**Figures 3C,D**), in accord with the immunoblotting results (**Figure 3B**). However, the sfGFP fluorescent signal level in *NRT*p was equivalent to or higher than that in *NIR*p (**Figures 3C,D**), in contrast to the result of immunoblotting (**Figure 3B**). The exact reason for this inconsistency is unclear at present, but it is likely that there are two sizes of sfGFP, and the larger one, which is specific to *NRT*p (**Figure 3B**), emits greater fluorescence. The sfGFP fluorescence was detectable for at least 2 weeks after the medium exchange (**Figures 3C,D**).

The above results indicate that the *NR, NIR*, and *NRT* promoters are inactive in the ammonium medium, with little evidence of leaky transcriptional activity, and are activated by the exchange of the ammonium medium for the nitrate medium. In addition, it has been shown that the transcriptional potency was the highest in the *NIR* promoter, followed by the *NRT, APCC* (a constitutive promoter) and *NR* promoters.

To further evaluate the strength of the transcriptional activity of the *NR, NIR*, and *NRT* promoters in the activated state (i.e., in the nitrate medium), we compared the mRNA levels of the *NR, NIR, NRT*, and *sfGFP* transgenes with other nucleus-encoded genes in *NIR*p cells grown in the nitrate medium by RNA sequence (RNA-seq) analysis. To compare mRNA level within a sample, the counts of the RNA-seq reads were normalized by the GC content bias correction method (Risso et al., 2011). The RNA-seq analysis detected transcripts of 4,740 genes among 4,774 *C. merolae* nucleus-encoded genes. It was ascertained that the *APCC* gene was very strongly transcribed in the cell in the nitrate medium (**Figure 3E**). The mRNA level was the highest in *sfGFP*, transcription of which was driven by the *NIR* promoter, followed by *NRT, NIR* and *NR* genes, whose ranking was the 59-th, 127-th, 352-th, and 1162-th highest among the nucleus-encoded genes, respectively (**Figure 3E** and Supplementary Data 1). It should be noted that strength of promoter activity and a corresponding mRNA level was not always consistent: the mRNA level of the *sfGFP* transgene was higher than that of the *NIR*, and the order of the promoter activities and the mRNA level of the *NIR* and *NRT* became inverse. It is likely that the *orf* sequence and the 3′ untranslated region are also involved in its mRNA turnover.

We further examined the effect of nitrate concentration and co-existence of ammonium and nitrate in the medium on promoter activity. QRT-PCR showed that the level of *sfGFP* mRNA increased depending on concentration of nitrate (**Figure 3F**), and that addition of 0.2 mM ammonium to the medium with 5 mM nitrate decreased the mRNA level (**Figure 3F**).

### Repression of the *NR, NIR*, and *NRT* Promoters in the Expression of the *sfGFP* Transgene by the Addition of Ammonium

**FIGURE 4:**
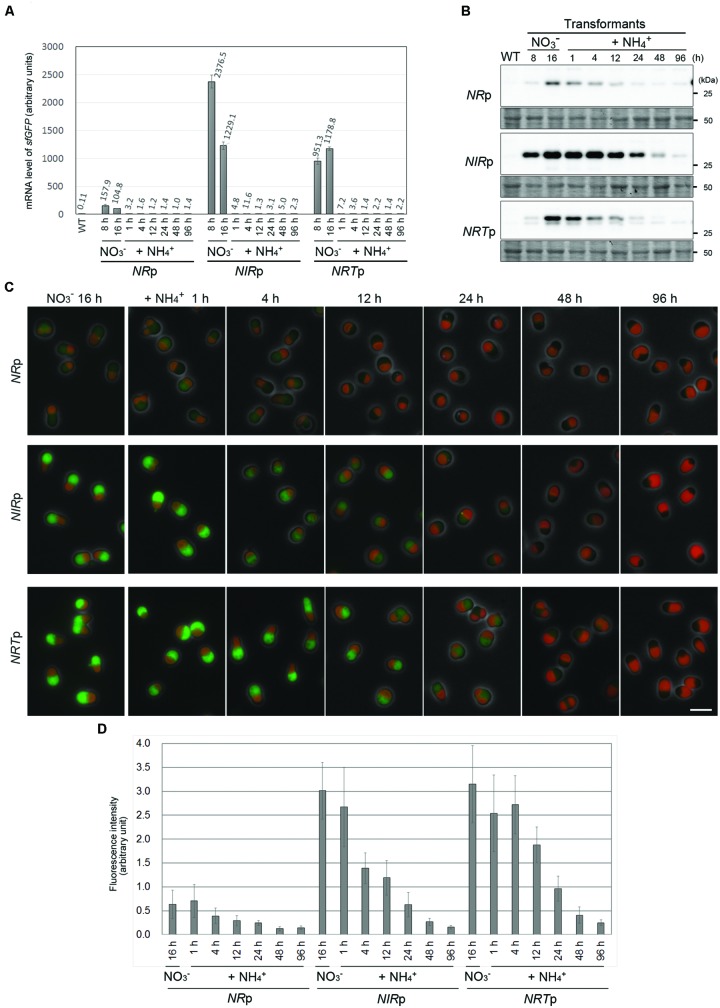
**The suppression of sfGFP expression by the addition of ammonium in the *NR*p, *NIR*p, and *NRT*p strains. (A)** Quantitative RT-PCR analyses showing the change in the *sfGFP* mRNA level in the *NR*p, *NIR*p, and *NRT*p strains before and after the addition of ammonium. The values in the respective stains were normalized with the data of *DRP3/*Cm*Dnm1*. The WT strain was used as a negative control. After cultivation of the cells in the nitrate medium for 16 h, ammonium was added to the medium and then the cells were cultured for 96 h. The bar indicates the standard deviation (*n* = 3). **(B)** Immunoblot analysis of the total cell lysates of the respective strains with the anti-GFP antibody showing the change in the sfGFP protein level before and after the addition of ammonium. An image of the PVDF membrane stained with Ponceau S is shown as a loading control. **(C)** Fluorescent micrographs showing the change in the level of the sfGFP fluorescent signal before and after the addition of ammonium. The exposure time to capture the sfGFP signal was 1 sec for all images. Green, GFP; red, autofluorescence of chlorophyll; gray, phase-contrast. The scale bar = 5 μm. **(D)** The change in the intensity of sfGFP fluorescence in cells in **(C)**. The bar indicates the standard deviation (*n* = 15).

The aim of this study was to develop a system that would be able to turn off, as well as turn on, the expression of genes of interest. To this end, we tested whether the expression of *sfGFP* was suppressed by adding ammonium to the culture of *NR*p, *NIR*p, and *NRT*p. After the cultivation for 16 h in the nitrate medium to express sfGFP constitutively, ammonium was supplied to the medium. qRT-PCR analysis showed that the mRNA level of *sfGFP* in each strain decreased to the basal level within 1 h after the addition of ammonium (**Figure 4A**). In contrast to the mRNA results, immunoblotting analysis and fluorescence microscopy showed that the level of the sfGFP protein gradually decreased and became hardly detectable at 96 h after the addition of ammonium (**Figures 4B–D**). These results showed that the transcription of a transgene driven by the *NIR, NR*, or *NRT* promoter effectively abrogated by the addition of ammonium.

## Discussion

In this study, we first evaluated the effectiveness of the two chromosomal loci of *C. merolae*, the intergenic region between CMD184C and CMD185C, and the upstream region of *URA5.3* as neutral loci for the expression of transgenes. The results showed that these loci have the same ability to express a transgene. Thus far, only the *URA5.3* selection gene marker has been available for the transformation of *C. merolae*. We are developing another marker. When the additional selection marker becomes available, multiple transgenes will be inserted into the two neutral chromosomal loci.

In addition, we have succeeded in developing an inducible and repressive transgene expression system in *C. merolae* by replacing nitrate (“on”) with ammonium (“off”) as the nitrogen source in the medium. The *NR, NIR*, and *NRT* promoters were constitutively activated in the nitrate medium, whereas the basal levels of the *NR, NIR*, and *NRT* promoter activity were extremely low in the presence of ammonium. This inducible system will be useful, as the previously developed heat-shock promoter system in *C. merolae*, for the expression of dominant-negative forms of proteins and the evaluation of the resultant primary defects in cells (Sumiya et al., 2014).

In terms of the repressive system, it will be useful for conditional gene knockdown, which has not been developed to date in photosynthetic eukaryotes. In this case, the *URA5.3* gene and *NR, NIR*, or *NRT* promoter will be inserted into the upstream region of a gene of interest (i.e., the endogenous promoter will be replaced with that of a nitrate-assimilation gene) in cells cultured in the nitrate medium. The formation of colonies after transformation will be feasible, because the sfGFP will have been continuously expressed at that time for a period of at least for 2 weeks, which is sufficient for colony formation (**Figure 3C**). Then, the expression of the gene of interest will be immediately turned off by the addition of ammonium to the medium. In this study, the level of sfGFP immediately decreased after the inactivation of the promoters by the addition of ammonium, but it took ∼96 h for sfGFP to reach the undetectable level (**Figure 4B**). However, because GFP is a very stable protein (Chalfie et al., 1994), it probably will take a much shorter period of time for a protein of interest to be degraded after the inactivation of the promoters in most cases.

For the conditional knockdown assays, it is preferable that the expression level of a gene of interest, the transcription of which is driven by a promoter of a nitrate-assimilation gene, is close to the expression level of the original endogenous promoter. In this regard, we have shown that the transcriptional activity level of the nitrate-assimilation genes varies and one may select the promoter according to the experimental design. To choose an appropriate promoter, the RNA-seq analysis performed in current study (**Figure 3E** and Supplementary Data 1). Even though the mRNA level does not always indicate strength of corresponding promoter, the data of the RNA-seq is useful for estimating and comparing the strength of the promoter of a target gene as well as the promoters of *NR, NIR*, and *NRT*. Based on the data of the RNA-seq (**Figure 3E**), the *NR* promoter will be applied to many genes exhibiting weak expressions whereas the *NIR* and *NRT* promoter will be applicable to highly expressional genes when conditional knockdown of a gene of interest is designed. In addition, it is possible to adjust promoter activity by changing nitrate and ammonium concentration (**Figure 3F**).

It is anticipated that the current results will extend the range of genetic analyses investigating gene function essential for cell survival, and will facilitate our understanding of the fundamental mechanisms of cell proliferation, organelle division and inheritance, environmental adaptation and related subjects in photosynthetic organisms.

## Material and Methods

### Cell Culture

The wild type *C. merolae* 10D (NIES-3377) along with the other strains were grown in 20 mL of MA2 medium (Ohnuma et al., 2008) in a tissue culture flask 25 (TPP Techno Plastic Products AG, Switzerland) with shaking at 130 rpm under continuous white light (50 μmol/m^2^s) at 40°C. The cultures of all of the strains were diluted to an OD750 of 0.2 in 60 mL of 2x Allen’s medium (Allen, 1959), incubated under continuous light (80 μmol/m^2^s) with aeration (400 mL ambient air/min) for 2 days and then collected for the experiments. 2x Allen’s medium, which contains (NH_4_)_2_SO_4_ as the sole nitrogen source, is termed the ammonium medium in this study. In order to induce the expression of *sfGFP* in the *NR*p, *NRT*p and *NIR*p strains, the medium was exchanged to a nitrate medium (NaNO_3_ 5 mM, Na_2_SO_4_ 20 mM, KH_2_PO_4_ 4 mM, MgSO_4_ 2 mM, CaCl_2_ 1 mM, twofold concentration of P4 metal, H_2_SO_4_ 0.03% v/v to adjust to pH 2.3) containing NaNO_3_ instead of (NH_4_)_2_SO_4_ as the sole nitrogen source (Imamura et al., 2008, with a minor modification). In order to suppress *sfGFP* transcription, 1 M (NH_4_)_2_SO_4_ solution was added to the nitrate medium at a final concentration of 20 mM 16 h after the medium exchange.

### Plasmid Construction and the Preparation of the Stable Transformants of *C. merolae*

The primers used in this study are listed in Supplementary Table S1. The U-*APCC*p strains were prepared as follows. The *URA5.3* (CMK046C) selection marker gene (*URA5.3 orf* flanked with 2,3-kb upstream and 1.9-kb downstream sequences) was amplified by PCR with primers No. 1 and No. 2 and *C. merolae* 10D genomic DNA as a template, and then cloned into the pGEM-Teasy vector (Promega) to prepare the pURA vector. To produce the pU-*APCC*p vector, the pURA vector was amplified by PCR with primers No. 3 and No. 4. The cassette containing *APCC* (CMO250C) promoter (the 600-bp upstream sequence flanking *APCC orf*), the *sfGFP orf* and the 200-bp downstream sequence flanking β-tubulin (CMN263C) *orf* was amplified by PCR with primers No. 5 and No. 6 and genomic DNA of the *C. merolae* D-*APCC*p stain (Sumiya et al., 2014) as a template, and then cloned into an amplified pURA vector using the In-Fusion HD Cloning Kit (Clontech). The resultant pU-*APCC*p vector was used as a template to amplify the assembled set of fragments (the ∼1.4-kb upstream sequence of *URA5.3 orf* [-2300 to -898], *APCC* promoter, *sfGFP orf*, 200-bp downstream sequence of β-tubulin *orf* and *URA5.3* selection gene [-897 to +471]) with primers No. 1 and No. 2.

The *NR*p, *NIR*p, and *NRT*p strains were prepared as follows. To prepare the pU-*APCC*p vector without the *APCC* promoter, the vector was amplified by PCR with primers No. 3 and No. 7. Then, the *NR* (CMG019C) promoter (the upstream flanking sequence of *NR orf* [-800 to 9 bp]), *NIR* (CMG021C) promoter (the upstream flanking sequence of *NIR orf* [-800 to 9 bp]) or *NRT* (CMG018C) promoter (the upstream flanking sequence of *NRT orf* [-800 to 9 bp]) was amplified by PCR with *C. merolae* 10D genomic DNA as a template and the primer set No. 8 and 9, No. 10 and 11, or No. 12 and 13, respectively. The respective promoters were cloned into the amplified pU-*APCC*p without the *APCC* promoter by using the In-Fusion HD Cloning Kit. The resultant p*NR*p::sfGFP, p*NIR*p::sfGFP, or p*NRT*p::sfGFP plasmid was used as a template to amplify the assembled set of fragments (the ∼1.4-kb upstream flanking region of *URA5.3 orf*, the promoter of *NR, NIR* or *NRT, sfGFP orf*, 200-bp downstream sequence of β-tubulin *orf* and the *URA5.3* selection gene with primers No. 1 and No. 2.

The respectively amplified DNA fragments for homologous recombination were transformed into *C. merolae* strain M4 (Minoda et al., 2004), a derivative of *C. merolae* 10D, which has a mutation in the *URA5.3* gene. Transformation was performed as described previously (Ohnuma et al., 2008; Imamura et al., 2010). A 4-μg aliquot of the PCR fragment was used for transformation and cells were incubated in MA2 medium containing uracil (0.5 mg/mL) for 1 day. After exchange to uracil-free MA2 medium, the cells were inoculated into starch on solidified MA2 under continuous white light at 42°C in ambient air supplemented with 5% CO_2_ until colonies were formed (for ∼2 weeks). The colonies were then transferred to starch on solidified MA2 medium containing uracil (0.5 mg/ml). Solidified MA2 medium was prepared according to Fujiwara et al. (2013b). The occurrence of the recombination events in the chromosomal *URA5.3* region in the U-*APCC*p, *NR*p, *NIR*p, and *NRT*p strains were confirmed by PCR with primers No. 14 and No. 15.

### Quantitative RT-PCR Analyses

Total RNA was extracted from a frozen cell pellet according to Fujiwara et al., 2009. cDNA was synthesized from 1 μg of the total RNA with an oligo(dT)_15_ primer using Prime Script Reverse Transcriptase (TAKARA Bio). Real-time PCR was performed using a StepOne Real-Time PCR System (Life technologies) in a 20-μl reaction mixture containing 4-μl template DNA, primers (0.25 nM each; Supplementary Table S1) and 10-μl Power SYBR Green Master Mix (Life technologies). Standard curves were constructed using serially diluted solutions of cDNA mixture prepared from the *NR*p, *NIR*p, and *NRT*p strains as well as the relevant sets of primers. The *sfGFP* mRNA level in the respective strains was normalized with the data of *DRP3/*Cm*Dnm1* (CME019C).

### Immunoblot Analysis

Fifteen μg of protein prepared from whole cell lysate were separated by SDS-PAGE, then transferred to a polyvinylidene difluoride (PVDF) membrane. The membrane was probed with an anti-GFP antibody (Abcam, ab6556) at a final concentration of 0.2 μg/ml, followed by goat anti-rabbit HRP-conjugated IgG (Vector Laboratories) at a dilution of 1:20,000. The chemiluminescent signals were detected with ECL Prime Western Blotting Detection Reagent (GE Healthcare) and a VersaDoc Imaging System (BIO-RAD).

### Fluorescence Microscopy

Images were captured using a fluorescence microscope (BX51; Olympus) with a digital CCD camera system DP71 (Olympus). The filter sets U-MNIBA3 and U-MWIG3 (Olympus) were used for the GFP fluorescence and auto-fluorescence of the chloroplast, respectively. The fluorescence intensity of sfGFP was quantified by ImageJ^1^.

### RNA-seq Analysis

Total RNA was extracted from the *NIR*p cells after cultivation for 24 h in the nitrate medium using Trizol reagent (Invitrogen) and RNeasy Mini Kit (Qiagen) according to the manufacturer’s protocol. mRNA was purified from 10 μg of total RNA of the *NIR*p cells using Dynabeads Oligo(dT)25 (Life technologies). Sequencing libraries were prepared by NEBNext mRNA library prep kit for Illumina (NEB) with following modifications. The oligo-dT primer was used for reverse transcription. After second strand synthesis, double stranded cDNA were fragmented to an average length of 300 bp using a Covaris S2 sonication system (Covaris, Woburn, CA, USA). One hundred cycles of paired-end sequencing were carried out using HiSeq2500 system according to the manufacturer’s specifications (Illumina). After the sequencing reactions were complete, the Illumina analysis pipeline (CASAVA 1.8.0) was used to process the raw sequencing data. The RNA-Seq reads were mapped to the *C. merolae* coding sequences^2^ using Bowtie2 with default parameters (Langmead and Salzberg, 2012). The Bowtie2 outputs were processed to obtain tag counts. Since it has been shown that the GC content can have a substantial impact on the read abundances in a RNA-Seq data set (Zheng et al., 2011), counts were full-quantile normalized within sample by the GC content bias correction methods implemented in the EDASeq R package (Risso et al., 2011). These normalized counts were used to calculate the expression level of each gene (in RPKM units) according to (Mortazavi et al., 2008).

## Author Contributions

Conceived and designed the experiments: TF, KT; performed the experiments: TF; analyzed the data: TF; contributed reagents/materials/analysis tools: AE, NS, KT; RNA-seq: YK, SH, HY; wrote the paper: TF, S-YM.

## Conflict of Interest Statement

The authors declare that the research was conducted in the absence of any commercial or financial relationships that could be construed as a potential conflict of interest.

## Footnotes

1 http://rsbweb.nih.gov/ij/download.html

2 http://merolae.biol.s.u-tokyo.ac.jp/download/cds_nt.fasta
