# Supplementary material for: A nitrogen source-dependent inducible and repressible gene expression system in the red alga Cyanidioschyzon merolae
Source: Front Plant Sci. 2015 Aug 26;6:657. doi: 10.3389/fpls.2015.00657 (PMC4549557; doi:10.3389/fpls.2015.00657)
Supplement: Supplementary file 1 [file Table_1.PDF]

Supplementary Table 1. Primers used in the current study.

No.	Primers	Sequence 5' to 3'
Primers used in cloning and transformation		
1	URA(-2300)F	CTTCAAGAAAAGAGGATCTTTTGCCGTGATGCC
2	URA(+471)R	CCCTAGCAGCTGACTGTATCTCTATTCTTAGGAAT
3	URA(-898)R	AGTCATACAACAGTACTCAGATCGTTGAGGAACAATGAAAG
4	URA(-897)F	GAACTGAGGGGCGAACGCAGTCCT
5	APCC(-600)Fura898	tactgttgtatgactCGACGAGAACGTATAAGGAGTGC
6	Bt3'(+200)Rura5'	ttcgcccctcagttcACACTTTTTGCCTGCACAAGT
7	sfGFP(1)F	ATGAGCAAGGGCGAGGAGCT
8	NR(-800)Fura898	tactgttgtatgactATGCACCATCATGCGTGCAT
9	NR(9)Rgfp	ctcgcccttgctcatAGTGTGCATCGTGTGGTACG
10	NIR(-800)Fura898	tactgttgtatgactCCGCTATCAATATCCGACGATATGCA
11	NIR(9)Rgfp	ctcgcccttgctcatGAACATCATCAGAGTATACCGCACG
12	NRT(-800)Fura898	tactgttgtatgactATTCGCGTCCCAGAGCGAG
13	NRT(9)Rgfp	ctcgcccttgctcatCTCCGCCATGCCGGAGT
Primers used to check for homologous recombination		
14	URA(-2400)F	TCCTAGCAGTTGCTCCAAACGTG
15	URA(+565)R	CCGGTCCGAATTCTGCGCT
Primers used in qRT-PCR analysis		
16	NR(2631)F	CTTTCAGTTCTGCTACGTACTGAG
17	NR(2700)R	AACGACAGCCTCCGGAAA
18	NIR(1911)F	TGTATTCTGGAAAAGCGCACG
19	NIR(2000)R	TGCGCATACGCCTCGAG
20	NRT(1501)F	AAGGACGATCTTGGTGTCTCG
21	NRT(1571)R	CGCCTATCGCTACGATCGG
22	DRP3(1981)F	GCAGGCGAAGTATCGAGC
23	DRP3(2050)R	AGTAACTCGCAAGAAGCGTCT
24	GFP(638)F	ACGAGAAGCGCGATCACA
25	GFP(717)R	CTTGTACAGCTCGTCCATGC

Lowercase letters indicate adaptor sequences for In-Fusion reaction.
